# Nanoscale Effects of Ethanol and Naltrexone on Protein Organization in the Plasma Membrane Studied by Photoactivated Localization Microscopy (PALM)

**DOI:** 10.1371/journal.pone.0087225

**Published:** 2014-02-04

**Authors:** Steven J. Tobin, Eliedonna E. Cacao, Daniel Wing Wo Hong, Lars Terenius, Vladana Vukojevic, Tijana Jovanovic-Talisman

**Affiliations:** 1 Department of Molecular Medicine, Beckman Research Institute of the City of Hope Comprehensive Cancer Center, Duarte, California, United States of America; 2 Department of Chemistry, University of Hawaii at Manoa, Honolulu, Hawaii, United States of America; 3 Department of Clinical Neuroscience, Karolinska Institutet, Stockholm, Sweden; Cornell University, United States of America

## Abstract

**Background:**

Ethanol affects the signaling of several important neurotransmitter and neuromodulator systems in the CNS. It has been recently proposed that ethanol alters the dynamic lateral organization of proteins and lipids in the plasma membrane, thereby affecting surface receptor-mediated cellular signaling. Our aims are to establish whether pharmacologically relevant levels of ethanol can affect the lateral organization of plasma membrane and cytoskeletal proteins at the nanoscopic level, and investigate the relevance of such perturbations for mu-opioid receptor (MOP) function.

**Methodology/Principal Findings:**

We used Photoactivated Localization Microscopy with pair-correlation analysis (pcPALM), a quantitative fluorescence imaging technique with high spatial resolution (15–25 nm) and single-molecule sensitivity, to study ethanol effects on protein organization in the plasma membrane. We observed that short (20 min) exposure to 20 and 40 mM ethanol alters protein organization in the plasma membrane of cells that harbor endogenous MOPs, causing a rearrangement of the lipid raft marker glycosylphosphatidylinositol (GPI). These effects could be largely occluded by pretreating the cells with the MOP antagonist naltrexone (200 nM for 3 hours). In addition, ethanol induced pronounced actin polymerization, leading to its partial co-localization with GPI.

**Conclusions/Significance:**

Pharmacologically relevant levels of ethanol alter the lateral organization of GPI-linked proteins and induce actin cytoskeleton reorganization. Pretreatment with the MOP antagonist naltrexone is protective against ethanol action and significantly reduces the extent to which ethanol remodels the lateral organization of lipid-rafts-associated proteins in the plasma membrane. Super-resolution pcPALM reveals details of ethanol action at the nanoscale level, giving new mechanistic insight on the cellular and molecular mechanisms of its action.

## Introduction

Alcohol is one of the most widely used and abused psychoactive substances, with a large negative effect on health and public safety. Despite considerable efforts, efficient pharmacotherapy for alcohol dependence is not yet available. This is partially explained by the fact that alcohol, unlike most other psychoactive drugs, does not act on one receptor system only, but rather modulates directly or indirectly a variety of neurotransmitter and neuromodulator systems.

Naltrexone, a general antagonist of opioid receptor function and the active substance in several clinically used drugs, was reported to reduce the relapse in heroin [1], alcohol [2], and amphetamine abuse [3], diminish the craving for food in obesity [4] and decrease hedonic responses in gambling [5]. While mechanisms underlying naltrexone action in treating opiate (heroin) abuse are well understood – naltrexone binding to opioid receptors blocks opiate binding to the receptor, the mechanisms underlying its effects in non-opiate abuse (such as alcohol and amphetamine), food abuse, and non-substance-related abuse are still not fully elucidated.

It is widely assumed that ethanol increases the activity of the endogenous opioid system through the release of opioid peptides and that anticraving/antihedonic effects of naltrexone are achieved through antagonizing the effect of opioid peptides acting at the mu-opioid receptor (MOP). However, this hypothesis is not fully validated experimentally [6]. Microdialysis studies have shown that high ethanol concentrations (>40 mM) increase extracellular endorphin levels in brain regions such as the nucleus accumbens [7] and the central nucleus of the amygdala [8], which seemingly supports the opioid surfeit hypothesis. However, microdialysis studies have also shown that aversive stimuli increase extracellular levels of β-endorphin in the nucleus accumbens [9], [10], suggesting that increased β-endorphin release in the nucleus accumbens may not necessarily reflect the rewarding and positive reinforcing effects of ethanol [6]. Furthermore, animal model studies have shown that MOP knockout mice do not self-administer alcohol [11], whereas neither null-mutation of preproenkephalin, nor homozygous knockout of proopiomelanocortin affects the voluntary intake of ethanol in mice [12]–[14]. Thus, modification of β-endorphin or enkephalin levels, which are the endogenous peptide ligands at MOP, does not affect the preference of ethanol intake in mice in contrast to the elimination of MOP [11]–[15]. Taken together, these results suggest that ethanol-induced surfeit of opioid peptides is not the only mechanism through which ethanol affects opioid signaling in the CNS, which brings into focus its actions at the receptor.

It has been recently proposed that ethanol can alter the lateral organization of proteins in the plasma membrane, thereby modulating the function of several cell-surface receptors including MOP [16]–[22]. The aim of this study is to establish whether pharmacologically relevant concentrations of ethanol (20 and 40 mM) change the lateral organization of plasma membrane and cytoskeletal proteins at the nanoscale level, and whether action of the opioid receptor antagonist naltrexone at endogenous MOPs is protective against such changes. To this end, we are using MDA-MB-468 cells that endogenously express opioid receptors [23], [24] and super-resolution fluorescence imaging by Photoactivated Localization Microscopy (PALM) [25], [26].

PALM and related pointillistic microscopy techniques [25]–[29] utilize switchable fluorescence reporters to enable single-molecule detection and localization with a precision of 15–25 nm, which is beyond the spatial resolution limit that is imposed by the diffraction of light. The advantage of PALM [25], [26] over other techniques with high spatial resolution, such as electron microscopy (EM) [30] or near field scanning optical microscopy (NSOM) [31], is the ability to observe proteins that are expressed with a fluorescent tag, such as photoactivatable Green Fluorescent Protein (paGFP) [32] or photoactivatable mCherry1 (pa-mCherry1) [33], which obviates all artifacts that are associated with covalent protein labeling and allows for minimally invasive sample handling. In addition, PALM is more versatile than EM, NSOM and Förster Resonance Energy Transfer (FRET) [34] because it enables us to study remodeling of cell surface protein organization with nanoscopic (15–25 nm) precision across the whole basal plasma membrane, rather than in a very small area. Furthermore, recent advances in data processing implemented by pair-correlation analysis (pcPALM) [35], [36] enable us to quantitatively characterize the lateral organization of proteins in the plasma membrane and quantify overall changes in protein density following the perturbation of the normal physiological state of the cell by treatment with bioactive compounds. Details of pcPALM analysis, equation derivations, and initial biological applications can be found in [35], [36]. We present here only basic outlines.

Spatial pair-correlation (pc) function describes the average probability of finding a molecule at a given distance from another molecule. pcPALM utilizes this statistical analysis approach to characterize distribution of proteins in PALM images and thus can quantitatively characterize the lateral distribution of molecules in the basal plasma membrane of cells. The overall pc function obtained by analysis of fluorescence distribution in pointillistic images has contributions from stochastic clustering, *i.e.* the multiple appearance of a single molecule due to blinking (stochastic auto-correlation, g_r_^stoch^) and the relative spatial distribution of protein molecules (protein auto-correlation, g_r_^prot^). The contribution of stochastic clustering can be easily identified because the multiple appearances of single molecules have a defined spatial distribution, and can thus be subtracted from the overall pc function to yield the protein correlation function [35], [36]. By fitting the protein correlation function using an exponential function, important lateral distribution parameters can be determined such as: the increased local density of proteins appearing in a cluster or domain (a unitless number), cluster radius, and number of detected proteins per cluster.

## Results

### Ethanol alters the lateral distribution of GPI-anchored proteins in the plasma membrane

**Figure 1:**
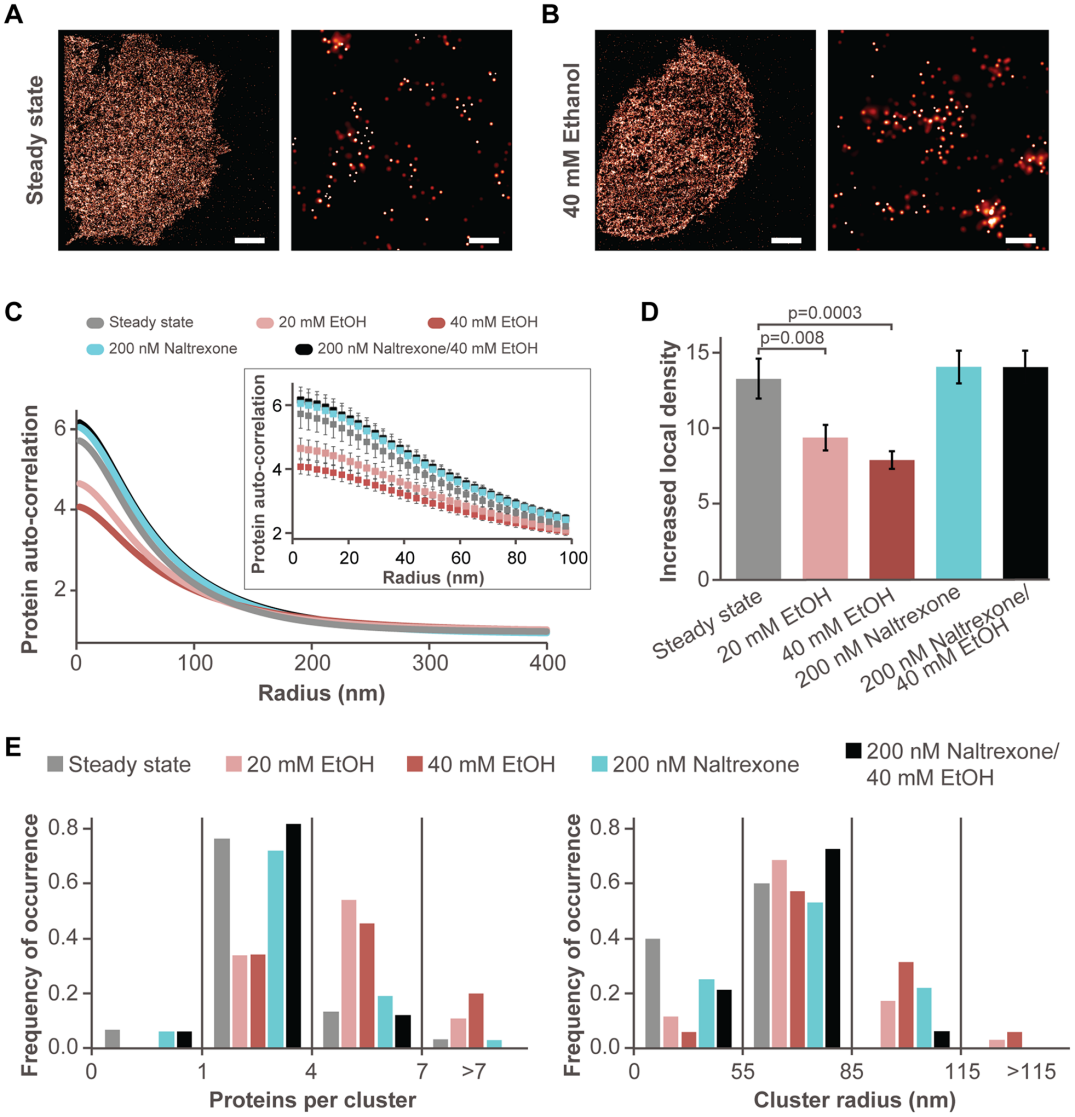
Effect of ethanol and naltrexone on GPI distribution. **A.** PALM image of the whole area (left, scale bar 5 µm) and a magnified area (right, scale bar 200 nm) showing GPI distribution in untreated MDA-MB-468 cells (control). **B.** PALM images of the whole area (left, scale bar 5 µm) and a magnified area (right, scale bar 200 nm) showing GPI distribution in MDA-MB-468 cells incubated with 40 mM ethanol for 20 min. Super-resolution images were generated by analyzing dataset using a standard PALM analysis [25]; peaks were grouped using maximum blinking time of 10 s for paGFP and group radius of 2.5 σ. Centers of peaks for panels A and B are given in Figure S1 in File S1 and Figure S2 in File S1. **C.** Average protein auto-correlation functions in untreated cells (gray, n = 31), after 20 min incubation in the presence of 20 mM ethanol (pink, n = 36), after 20 min incubation in the presence of 40 mM ethanol (russet, n = 36), upon 20 min incubation with 200 nM naltrexone (cyan, n = 33) and upon 3 h preincubation with 200 nM naltrexone followed subsequently by a 20 min incubation in the presence of 40 mM ethanol (black, n = 34). Insert shows magnified area at shorter radii with s.e.m. **D.** Increased local density of paGFP-GPI with s.e.m. Ethanol addition significantly decreased local density of GPI compared to that in untreated cells. **E.** Distribution of paGFP-GPI molecules per cluster (left) and cluster radius (right). Ethanol addition had significant increase in number of detected proteins per cluster and cluster radius compared to steady state, whereas no significant change is observed for other perturbation conditions.

Lateral distribution of the lipid raft marker glycosylphosphatidylinositol-anchored protein tagged with paGFP (paGFP-GPI) was investigated in MDA-MB-468 cells transiently transformed to express paGFP-GPI. To investigate the influence of bioactive compounds on GPI distribution in the basal plasma membrane, we imaged MDA-MB-468 cells expressing paGFP-GPI before and after addition of small molecules (Figure 1A and B). We analyzed multiple cell regions (as described in Materials and Methods) and determined the local cluster density, cluster radius, and number of detected proteins per cluster for each region. We show average protein auto-correlation functions (Figure 1C), average increased local cluster density (Figure 1D), the distribution of number of detected proteins per cluster (Figure 1 E, left) and the distribution of cluster radii (Figure 1E, right), to investigate if there is significant protein redistribution upon addition of a bioactive compound. Ethanol effects were evaluated after 20 min incubation with 20 mM or 40 mM ethanol. The effects of naltrexone alone were evaluated after 20 min incubation with 200 nM naltrexone. The protective effect of naltrexone against alcohol action was evaluated by pretreating the cells for 3 h with 200 nM naltrexone, washing, and then incubating with 40 mM ethanol for 20 minutes.

pcPALM data suggest that acute, 20 min long incubation with 20 mM and 40 mM ethanol alters GPI distribution in the plasma membrane (Figure 1, Figure S1 in File S1 and Fig. S2 in File S1). To assess the extent of ethanol action on GPI distribution, we first generated average protein correlation functions (Figure 1C) from the overall auto-correlation curves. These functions contain information about GPI distribution and do not include stochastic clustering effects [35], [36]. The overall auto-correlation functions with fits, stochastic clustering contribution, and protein clustering contribution for paGFP-GPI are given in Figure S3 in File S1. Data in Figure 1C indicate that addition of ethanol (20 mM in pink and 40 mM in russet) produces a significant perturbation of GPI distribution from the steady state before treatment with ethanol (gray), and suggests that the perturbation of GPI distribution may be dependent on ethanol concentration. To further analyze our data, we used pc analysis to obtain quantitative information on GPI distribution. Upon acute exposure to ethanol, we observed larger clusters with a higher number of detected GPI proteins and lower local density of proteins compared to the clusters present in steady state (Figure 1D and E). We also observed that ethanol induces a higher average density of GPI molecules (Figure S4A in File S1). A similar effect is observed with MOP receptors in FCS experiments [21]. We have verified that the average number of multiple appearances of a single paGFP molecule (contributing to stochastic auto-correlation) is comparable for the steady state and the ethanol-treated cells, and that dense regions do not have a significant impact on the average protein auto-correlation function (Fig. S4B in File S1). Moreover, correlation between protein density and the number of detected peaks or cluster radius was not observed (data not shown). The 20 min incubation with pharmacologically relevant levels of naltrexone (200 nM) has a negligible effect on GPI organization (Figure 1C–E). Interestingly, a 3 h pretreatment with the same concentration of naltrexone followed by 20 min incubation with 40 mM ethanol largely prevented GPI redistribution upon acute ethanol treatment. Average protein correlation functions for GPI in steady state, upon naltrexone treatment, and upon ethanol treatment with naltrexone preincubation showed very similar distributions (Figure 1C–E).

### Ethanol induces actin polymerization and partial co-localization with GPI-anchored proteins

**Figure 2:**
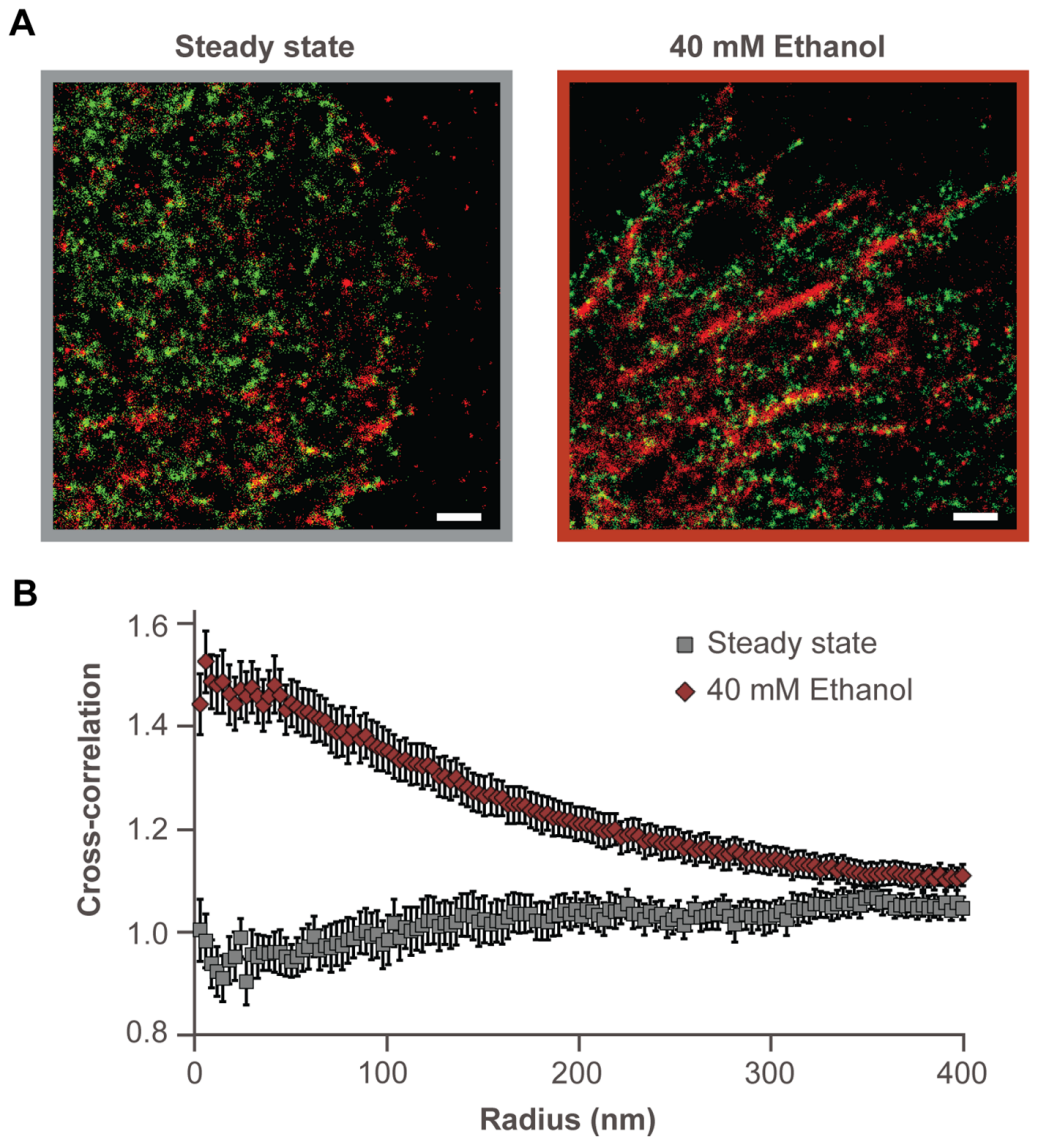
Differences in the distribution of paGFP-GPI and pa-mCherry1-actin, upon ethanol addition can be quantified using pcPALM. **A.** Section of a MDA-MB-468 cell coexpressing paGFP-GPI (green) and pa-mCherry1-actin (red) in the absence of ethanol (lined in gray) and upon addition of 40 mM ethanol (lined in russet). Centers of peaks are shown. Scale bars 1 μm. **B.** The cross-correlation curve indicates that actin and GPI were uncorrelated in the steady state (gray squares, s.e.m, n = 24, c(r) ∼1). However, c(r) increased following ethanol addition (russet diamonds, s.e.m, n = 27) indicating partial spatial co-localization between actin and GPI.

To investigate the effect of acute exposure to ethanol on actin organization and its distribution in the plasma membrane with respect to GPI, MDA-MB-468 cells transiently transformed to express paGFP-GPI and pa-mCherry1-actin were incubated with 40 mM ethanol for 20 min. Pronounced actin polymerization was observed upon incubation with ethanol (Figure 2A), which lead to partial GPI-actin co-localization. This is evident from the cross-correlation curve that builds-up after treatment with ethanol (Figure 2B, russet diamonds) but is not observed in the steady state, before treatment with alcohol (Figure 2B, gray squares).

### Endogenous MOP shows non-uniform lateral distribution and partial co-localization with GPI

**Figure 3:**
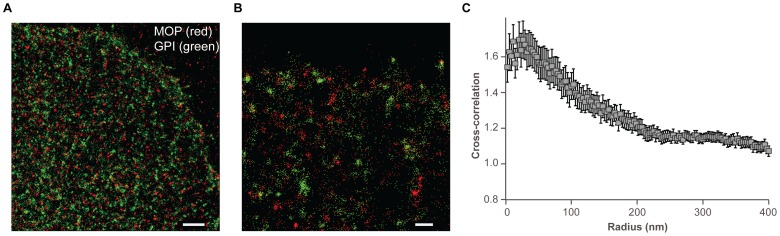
Distribution of MOP and GPI. **A.** Section of an untreated MDA-MB-468 cell (scale bar 2 µm) and **B.** Magnified area (scale bar 500 nm) showing distribution of antibody detected MOP (in red) and paGFP-GPI (in green). Centers of peaks are shown. PALM images showing distribution of antibody detected MOP (no expression of paGFP-GPI) are given in Figure S5 in File S1. **C.** The cross-correlation curve indicates that MOP and GPI show partial co-localization in the steady state (gray squares, s.e.m, n = 23), suggesting that a fraction of MOP likely resides in GPI-enriched lipid rafts.

Immunocytochemistry was used to visualize the endogenously expressed MOP in unmodified MDA-MB-468 cells. Single-molecule detection and spatial localization by PALM revealed that detected MOP molecules are not homogeneously distributed in the plasma membrane, showing regions of MOP accumulation interspaced with regions of comparatively low local MOP density (Figure S5 in File S1). Two-color pair cross-correlation PALM was used to characterize the local distribution of antibody against MOP and GPI molecules relative to each other in MDA-MB-468 cells transiently transformed to express paGFP-GPI (Figure 3). Distribution of endogenous MOP detected using antibody is shown in red and distribution of lipid raft marker GPI is shown in green. Images point out to partial spatial overlap of these two proteins in some regions. To quantitatively assess the co-localization, we conducted cross-correlation analysis. A pronounced peak in the pair cross-correlation curve, g(r)  = 1.7 at ∼30 nm, indicated that the pairwise spatial arrangement was not random, suggesting that detected MOP and GPI co-localize at spatial scales below the diffraction limit of classical fluorescence microscopy. Two-color pair cross-correlation PALM thus revealed that a fraction of MOP likely resides in GPI-enriched lipid rafts.

## Discussion

Ethanol affects the signaling of several important neurotransmitter and neuromodulator systems in the CNS, but the detailed mechanisms of its action at the molecular and cellular level remain still unknown. This lack of basic knowledge and, in particular, quantitative data continues to be a significant limitation when designing new drugs for the treatment of alcoholism. The question whether ethanol evokes its effects by acting on lipids or proteins is still debated [37]. Early work focused on ethanol interactions with membrane lipids, suggesting that ethanol alters plasma membrane fluidity, curvature, and lipid phase transitions [38]. Electrophysiology shifted the focus to ethanol interactions with plasma membrane proteins, primarily ligand-gated ion channels such as GABA, NMDA and AMPA, glycine or nicotinic acetylcholine receptors, suggesting that ethanol can bind to a pocket in certain subunits, thereby altering the sensitivity of the receptor to its ligand [39]. More recently, structural work identified ethanol interactions with a motif in the proton-activated *Gloeobacter violaceus* ligand-gated ion channels [40]. In either case, concentrations of ethanol needed to produce the proposed changes have been much higher than those commonly reached *in vivo* [41].

Several studies have indicated that ethanol may affect surface receptor signaling by affecting the lateral organization of proteins and lipids in the plasma membrane [16]–[22]. The cholesterol-reducing effect of ethanol [42]–[44] may alter the dynamics/structure of lipid rafts, thus perturbing the local nano-environment of cell surface proteins and their signaling. Our PALM studies on breast carcinoma MDA-MB-468 cells show that this is indeed true and that short-term exposure (20 min) to 20 mM and 40 mM ethanol alters protein organization in the plasma membrane, causing a rearrangement of the lipid raft marker GPI (Figure 1). This reorganization could potentially point out to loss of protein partners, such as MOP, in lipid rafts. In addition, ethanol induced pronounced actin polymerization (Figure 2) leading to partial co-localization of two important organizing domain markers, GPI and actin.

Our studies also show that pretreatment of MDA-MB-468 cells with 200 nM naltrexone is protective against ethanol-induced rearrangement of the lipid raft marker GPI (Figure 1). MDA-MB-468 cells endogenously express MOPs, as shown in Figure 3, Figure S5 in File S1, and also by Drell et al. [23] and Entschladen et al. [24]. The mechanisms through which the protective effects of naltrexone are achieved are not well known. It was shown previously that the carboxyl tail of the human MOP binds the carboxyl terminal region of human filamin A, a protein known to couple membrane proteins to actin [45], [46]. Interactions with filamin A have been noted for a range of other GPCRs and ion channels and have been shown to alter the trafficking properties of the interacting GPCRs (recently reviewed in [47]–[49]). We hypothesize that in the presence of ethanol, MOP-filamin A interactions may be disrupted, enhancing the fraction of opioid receptors that are not bound to filamin A/actin. Decoupling of MOP from filamin A and actin may lead to its increased lateral mobility, as was previously observed by FCS [21]. In addition, actin polymerization may reduce the area that is available for MOP free diffusion, further contributing to the previously observed shift of the auto-correlation function to shorter characteristic times [21].

It was recently shown that naltrexone can bind to filamin A with very high affinity [50], [51], a finding that still needs to be replicated. However, it is interesting to contemplate that the dual action of naltrexone on MOP and filamin A may stabilize ternary complexes of MOP, filamin A, and G proteins. MOP association in these complexes may explain the decrease in MOP lateral mobility that was previously observed by FCS [21]. Thus, pretreatment with naltrexone may lead to the formation of a larger scaffolding area that is less sensitive to ethanol-induced perturbations, thereby exerting a protective effect against ethanol action.

### Conclusions

pcPALM shows that acute (20 min) exposure to pharmacologically relevant concentrations of ethanol (20 mM and 40 mM) causes lateral reorganization of plasma membrane and cytoskeletal proteins in the investigated cellular model. In particular, ethanol-induced remodeling of MDA-MB-468 plasma membrane organization involves redistribution of the lipid raft marker GPI and pronounced actin polymerization. We cannot say, as yet, whether the observed effects of ethanol are specifically mediated through the endogenously expressed MOPs, but we have shown that pretreatment with the MOP antagonist naltrexone is protective against ethanol-induced plasma membrane remodeling.

Quantitative super-resolution imaging techniques can provide information about nanoscale spatial organization and are increasingly used to elucidate the mechanisms of various biological processes [36], [52]–[56]. Our data clearly show that pcPALM is a superb tool for investigation of protein distribution and co-localization at a nanoscale level and suggest that both plasma membrane lipid environment and actin cytoskeleton potentially play an important role in molecular mechanisms of alcohol action.

## Materials and Methods

25-mm #1.5 coverslips (Warner Instruments) were cleaned with 1% Hellmanex III (Fisher Scientific) for 3 h, followed by distilled water and 100% ethanol. Cleaned coverslips were subsequently flamed and placed in sterile 35-mm tissue culture dishes. For PALM microscopy, cells were grown on coverslips coated with fibronectin-like engineered protein (25 µg/ml in PBS, pH 7.4, Sigma). MDA-MB-468 and COS-7 cells (originally obtained from the American Type Culture Collection, ATCC) were cultured in Phenol Red-free Dulbecco's Modified Eagle Medium (DMEM) supplemented with 10% fetal bovine serum, 1 mM sodium pyruvate, 100 units/ml penicillin, 100 units/ml streptomycin, and 2 mM L-alanyl-L-glutamine. GPI and β-actin constructs were transiently transfected in MDA-MB-468 cells using Jetprime (PolyPlus) transfection reagent per manufacturer's instructions. Vesicular stomatitis virus glycoprotein tagged with paGFP (VSVGtsO45-paGFP) construct was transiently transfected in COS-7 cells using Jetprime (PolyPlus) transfection reagent per manufacturer's instructions and incubated at 32°C for at least 8 h prior to fixation. Approximately 24–36 h after transfection, the cells were washed quickly in phosphate buffered saline (PBS) pH = 7.4 at 37°C and fixed in 4% (w/v) paraformaldehyde and 0.2% (w/v) glutaraldehyde (Electron Microscopy Sciences) for 30 min at room temperature in PBS. These fixation conditions have been reported to immobilize most of the plasma membrane proteins [57]. Quenching was done with filter-sterilized 25 mM glycine in PBS for 10 min, and cells were finally washed three times with PBS. Coverslips were incubated with 1∶4000 diluted TetraSpeck beads (Invitrogen) in PBS for 5 min that served as fiducial markers to correct for drift during image acquisition and to overlay two-color images. Coverslips were imaged immediately after preparation in Attofluor cell chambers (Invitrogen) supplemented with PBS.

To investigate the effect of ethanol and naltrexone on GPI distribution we have: **a)** Supplemented the fresh cell culture medium with 20 mM or 40 mM ethanol, incubated the cells for 20 min at 37°C and fixed them as described above. **b)** Supplemented the fresh cell culture medium with 200 nM naltrexone, incubated the cells for 20 min at 37°C and fixed them; **c)** Supplemented the fresh cell culture medium with 200 nM naltrexone and incubated the cells for 3 h at 37°C, exchanged the cell culture medium with a fresh medium supplemented with 40 mM ethanol, incubated the cells for 20 min at 37°C and fixed them.

Primary anti-mu opioid receptor antibody (guinea pig polyclonal), and secondary rabbit anti-guinea pig IgG antibody were purchased from Abcam. The secondary antibody was labeled with Cage 552 (Abberior). A 1∶10 (v/v) solution of 10 mg/ml of dye dissolved in dimethyl sulfoxide (DMSO) and 2 mg/ml secondary antibody in PBS pH 7.4 with 0.1 M NaHCO_3_ was mixed and allowed to react for 2 h. Solution was quenched with 1.5 M hydroxylamine (pH 8.5). Unconjugated dye was removed by passing the solution through a size exclusion chromatography column (Bio-Rad). Prior to experiment, labeled antibody was passed through 300 kDa concentrator to remove any potential aggregates. Concentration of labeled secondary antibodies was measured by Bradford assay. Immunochemistry was done according to established protocols. Briefly, cells were fixed for 20 min at room temperature with 4% (w/v) paraformaldehyde and 0.2% (w/v) glutaraldehyde and inactivated with 25 mM glycine for 5 min. After 3 washes in PBS, cells were incubated in permeabilization buffer (PB, 0.5% tween-20, 5% BSA in PBS) for 20 min. After PBS wash, cells were incubated for 1 h in PB supplemented with 2 μg/ml of primary antibody. Subsequently, cells were washed and incubated with 5 μg/ml of Cage 552-labeled secondary antibody in PB for 45 min. After another PBS wash, cells were fixed for 10 min with 4% (w/v) paraformaldehyde and 0.2% (w/v) glutaraldehyde, and inactivated with 25 mM glycine for 10 min at room temperature.

PALM imaging was performed on Nikon Instruments Ti-E inverted microscope with a 60×/1.45 NA TIRF objective (Plan Apo); 405 nm (100 mW OBIS, Coherent), 488 nm (100 mW Sapphire, Coherent) and 561 nm (50 mW Sapphire, Coherent) lasers in a 4-Laser Module (Nikon) with EM-CCD camera (Andor Technology, iXon DU897) and DD12NLC 1.2 X C-Mount (SPOT Imaging Solutions). Images of a 38×38 μm^2^ area were collected with an exposure time of 100 ms. paGFP was simultaneously activated and excited with 488 nm laser with the intensity set to 2.9 mW (as measured at rear aperture of the objective). For two-color imaging, paGFP fluorescence was collected first by activating/exciting with 488 nm laser until paGFP was completely exhausted. Next pa-mCherry1 fluorescence was collected using 405 nm (150 µW), and 561 nm (2.8 mW) lasers for activation and excitation, respectively; Cage 552 fluorescence was collected using 405 nm (350–400 µW), and 561 nm (2.8 mW) lasers for activation and excitation, respectively.

Peaks were localized using a previously described algorithm written in IDL (Research Systems, Inc.) [25]. Identified peaks were fit using a cylindrically symmetric Gaussian point spread function. All the detected peaks that appear in successive frames and area within a radius of 2.5× sigma (σ) are grouped as a single peak. These grouped peaks are used for subsequent pcPALM analysis using code custom written in MATLAB (The Mathworks, Inc., Natick, MA). Binary images of cells were generated using the grouped peak coordinates where the detected peaks are assigned a value of 1, while the rest have a value of 0. For dual-color imaging, two separate binary images were generated for two different proteins. The mean localization of detected single peak was estimated from the distribution of sigma of grouped peaks; average sigma was generally around 20 nm and peaks with sigma greater than 35 nm were discarded. Average number of appearance of individual paGFP molecules (due to blinking) was estimated to be equal to 9. This number was calculated based on the image analysis of 1) sparse paGFP covalently attached on the surface (n = 300 paGFP molecules) and 2) paGFP-GPI sparsely expressed in MDA-MB-468 cells in steady state and upon 40 mM acute ethanol treatment (n = 18 paGFP molecules for both conditions). Pair-correlation (auto- and cross-correlation) was computed on selected regions of the cell using Fast Fourier Transforms. To further validate the imaging and processing parameters, we investigated distribution of trimeric VSVGtsO45-paGFP in COS-7 cells using the same acquisition and processing conditions. We obtained cluster radius of less than 65 nm (92%), with average of 3 detected proteins per cluster and increased local density of 31.7±5.5 for regions of 4–16 µm^2^ (n = 12, data not shown).

For both single-color data (auto-correlations of GPI) and dual-color data (cross-correlations of GPI-actin and GPI-MOP), square regions of 16 μm^2^ were analyzed. Minimum number of analyzed cells for single-color GPI imaging was 15, while the number of analyzed cells for dual-color imaging of GPI-actin and GPI-MOP were 12 and 6, respectively. Results of pc analysis for single-color data are presented in Figure 1. All parameters presented in this figure (cluster radius, number of detected proteins per cluster and increased local density of proteins in the cluster) are fitted to the exponential function with R^2^ ≥0.95. Error bars represent standard error of the mean (s.e.m.) for untreated cells (n = 31), 20 mM ethanol-treated cells (n = 36), 40 mM ethanol-treated cells (n = 36), 200 nM naltrexone-treated cells (n = 33) and cells preincubated with 200 nM naltrexone followed by 40 mM ethanol (n = 34). Statistical significance (p<0.01) of increased local density was analyzed using Student's t-test (one-tailed distribution with two-sample unequal variance). For the dual-color data, average cross-correlation curve is presented with s.e.m (error bars) for steady state (n = 24) and upon addition of 40 mM ethanol (n = 27) of GPI-actin in Figure 2B, and for steady state of GPI-MOP (n = 23) in Figure 3C.

## Supporting Information

File S1 **Contains supporting Figures S1-S5 with legends.**
(DOC)
