# Supplementary material for: Nanoscale Effects of Ethanol and Naltrexone on Protein Organization in the Plasma Membrane Studied by Photoactivated Localization Microscopy (PALM)
Source: PLoS One. 2014 Feb 4;9(2):e87225. doi: 10.1371/journal.pone.0087225 (PMC3913589; doi:10.1371/journal.pone.0087225)
Supplement: File S1 — Contains supporting Figures S1-S5 with legends. (DOC) [file pone.0087225.s001.doc]

**Supporting Information**

**Nanoscale effects of ethanol and naltrexone on protein organization in the plasma membrane studied by Photoactivated Localization Microscopy (PALM)**

Steven J. Tobin, Eliedonna E. Cacao, Daniel Wing Wo Hong, Lars Terenius, Vladana Vukojevic, Tijana Jovanovic-Talisman

**
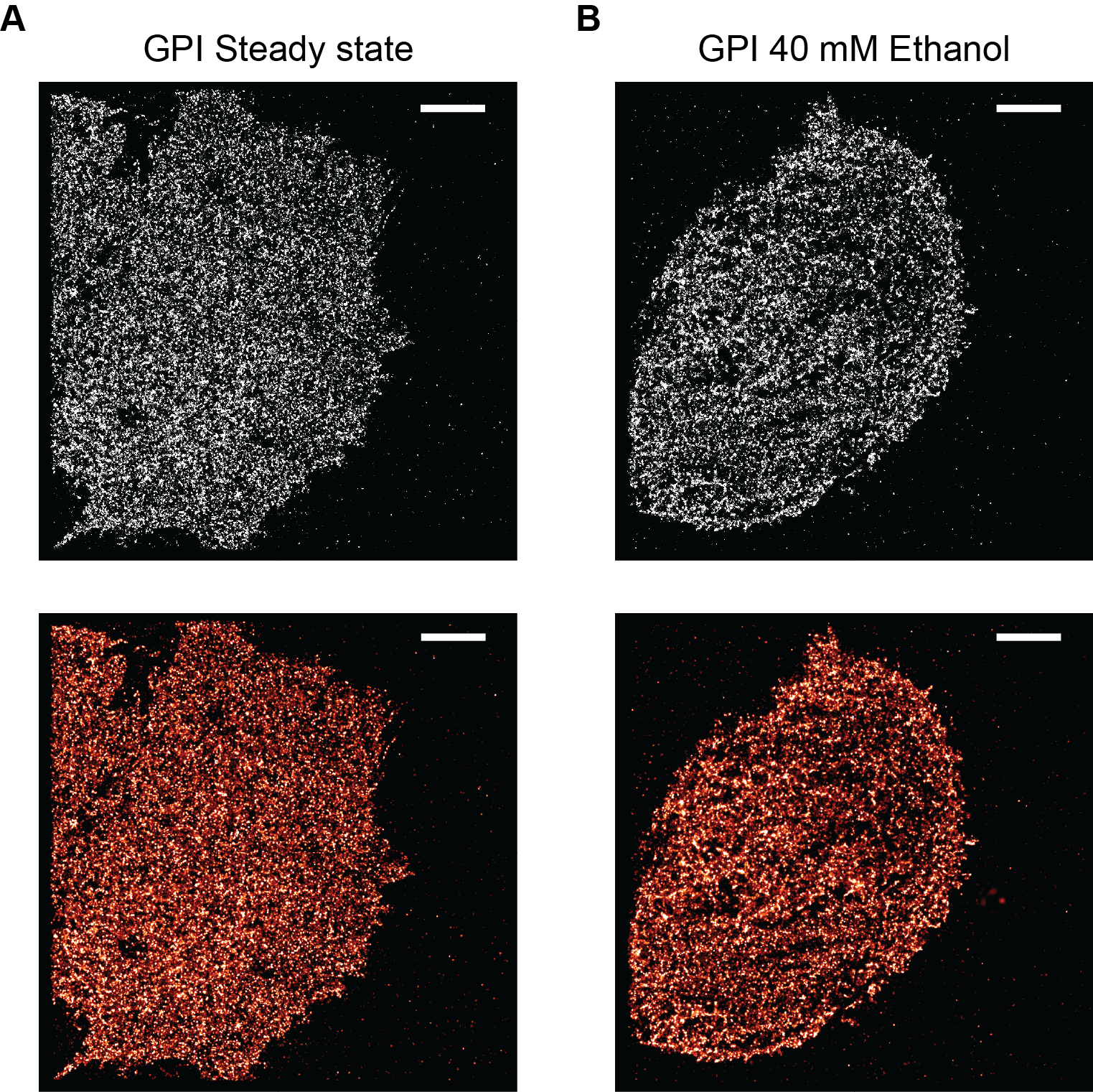
**

**Figure S1. Ungrouped PALM images of the whole basal membrane of MDA-MB-468 cells.** Centers of peaks (top) and rendered peaks (bottom) are shown for images of the whole area presented in the main text in Figure 1. **A.** GPI in steady state **B.** GPI upon 40 mM ethanol treatment**.** All scale bars are 5 μm.

**
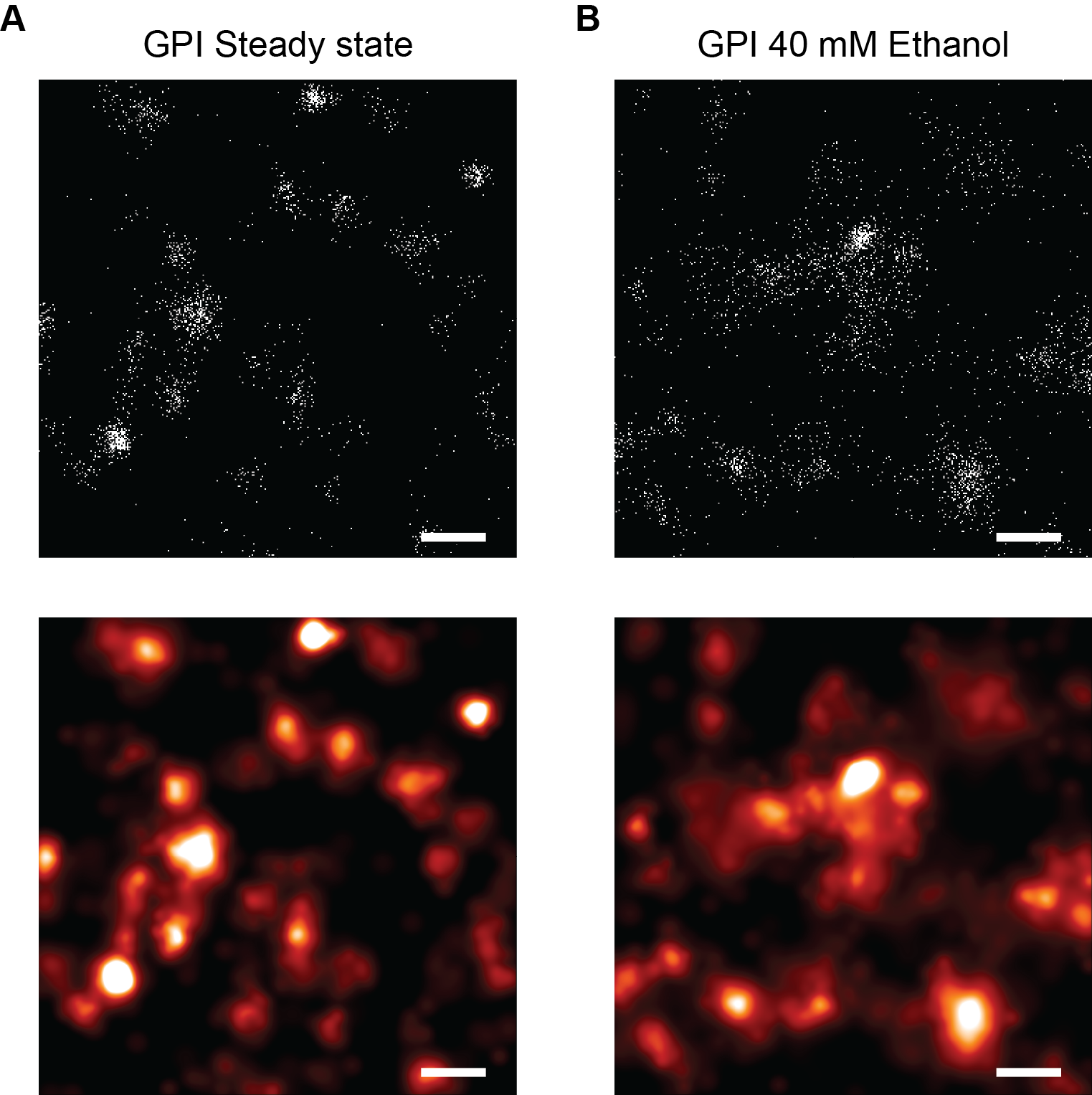
**

**Figure S2. Ungrouped PALM images of the zoomed regions.** Centers of peaks (top) and rendered peaks (bottom) are shown for zoomed images presented in the main text in Figure 1. **A.** GPI in steady state **B.** GPI upon 40 mM ethanol treatment. All scale bars are 200 nm.

**
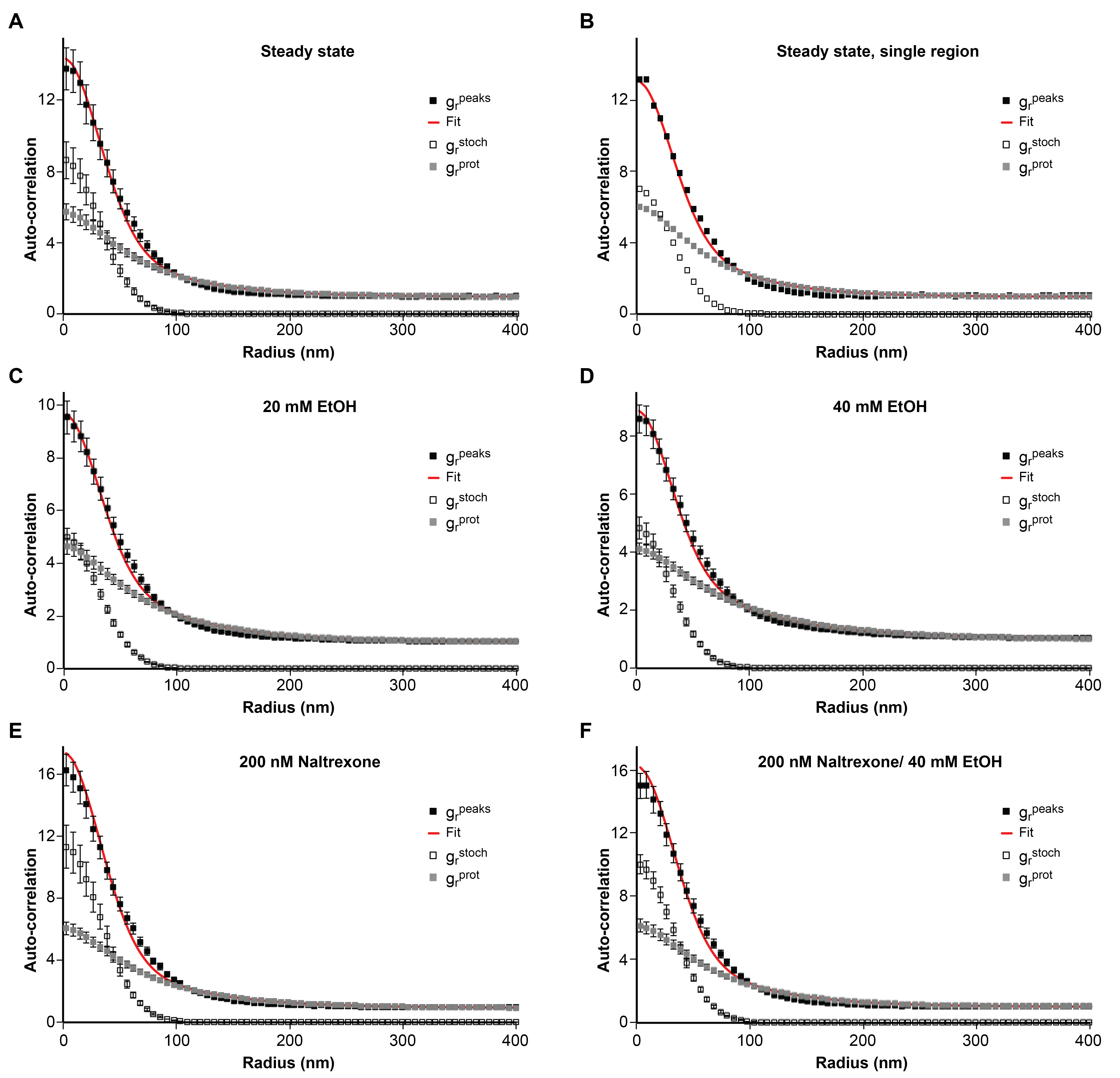
**

**Figure S3.** The overall auto-correlation function (g_r_^peaks^) has contributions from stochastic clustering (g_r_^stoch^) and the relative spatial distribution of protein molecules (g_r_^prot^). Fit to clustered model (red line) of the correlation functions for cells expressing paGFP-GPI **A.** in steady state (R^2^ =0.97, s.e.m., n=31); **B.** in steady state (R^2^ =0.98, n=1); data for one representative area are shown; **C.** upon addition of 20 mM ethanol (R^2^ =0.97, s.e.m., n=36); **D.** upon addition of 40 mM ethanol (R^2^ =0.97, s.e.m., n=36); **E.** upon addition of 200 nM naltrexone (R^2^ =0.96, s.e.m., n=33); **F.** upon preincubation with 200 nM naltrexone followed by addition of 40 mM ethanol (R^2^ =0.95, s.e.m., n=34). Correlation due to multiple appearances of a single protein (g_r_^stoch^) and protein correlation (g_r_^protein^) are calculated from the fit parameters. Values for increased local density, cluster radius, and number of detected proteins per cluster are calculated from individual areas such as one given in panel B.

**
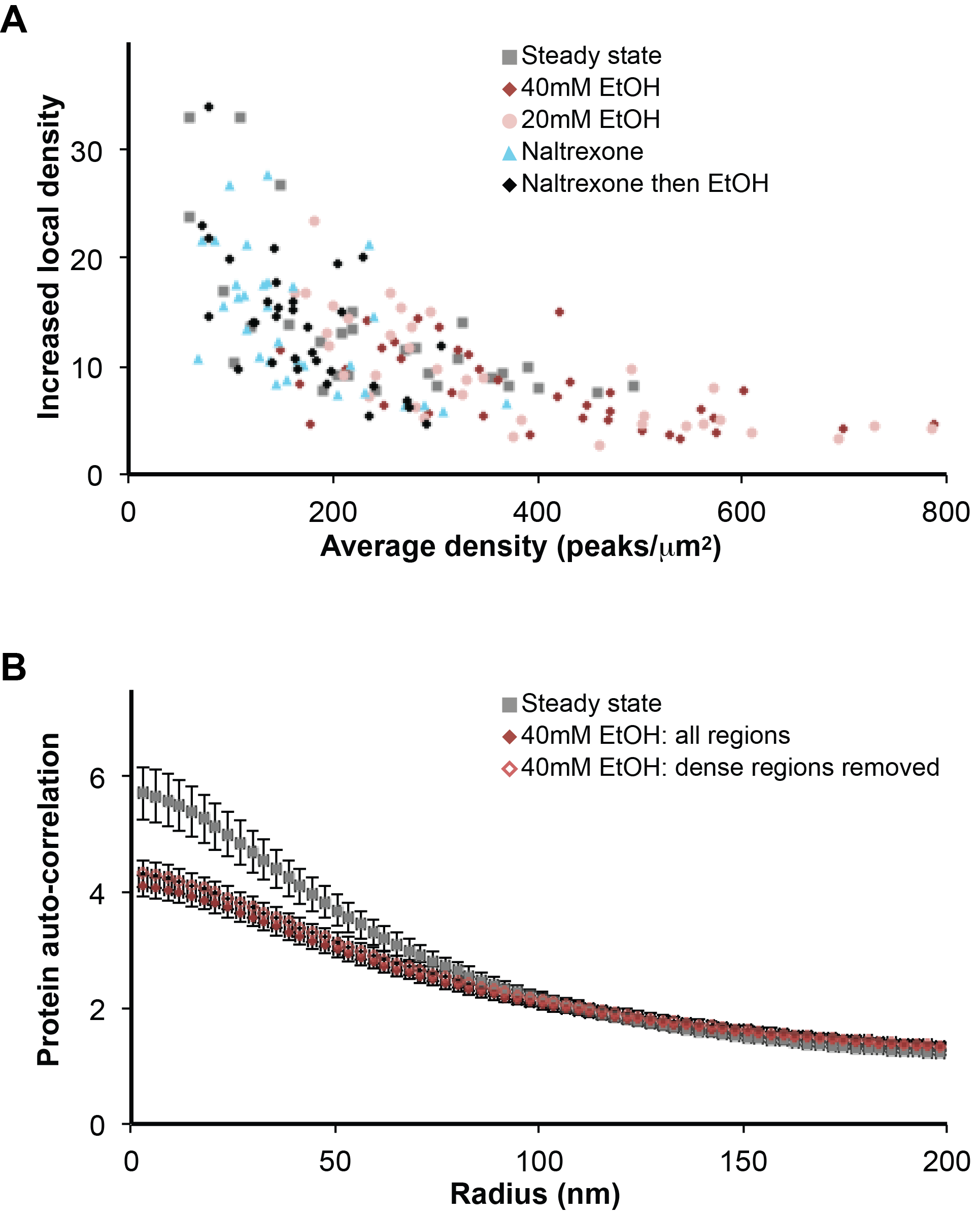
**

**Figure S4. Average density appears to be increased upon ethanol treatment.** **A.** Increased local density is plotted vs. average density (number of detected peaks per area). Ethanol induces somewhat higher average density. Average density does not appear to correlate with number of detected proteins per cluster or cluster radius. **B.** Average protein auto-correlation functions for steady state (gray squares, s.e.m., n = 31), after 20 min incubation in the presence of 40 mM ethanol (filled russet diamonds, s.e.m., n = 36), and after 20 min incubation in the presence of 40 mM ethanol with dense areas removed (open pink diamonds, s.e.m., n = 30) are shown. Higher density regions detected upon ethanol treatment did not have a significant effect on the average protein auto-correlation functions.

**
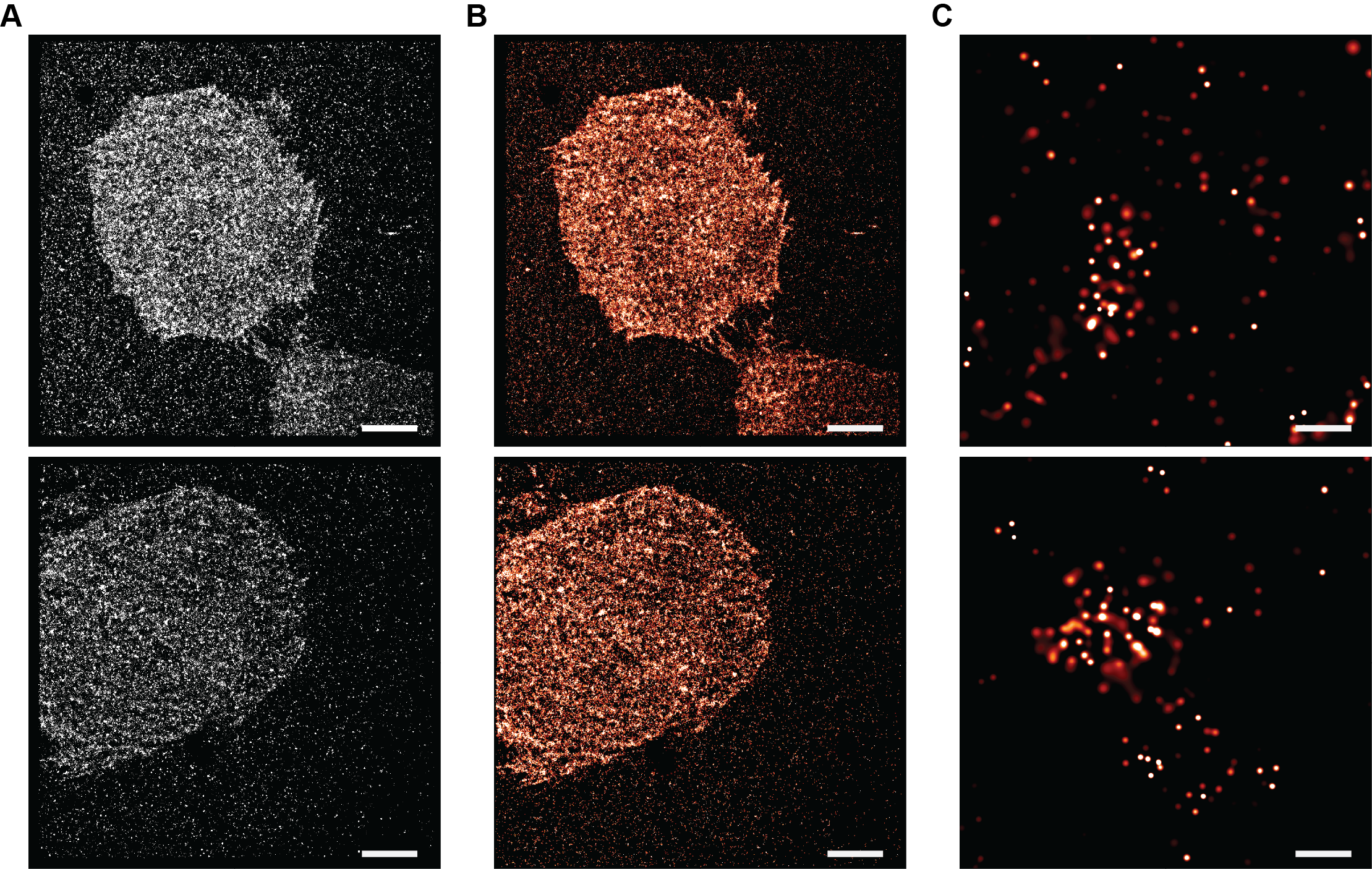
**

**Figure S5. MOP distribution.** Image of the whole area (**A, B**, scale bar 5 µm) and a magnified area (**C**, scale bar 200 nm) showing distribution of antibody detected MOP in untreated MDA-MB-468 cells. Super-resolution images were generated by analyzing the dataset using a standard PALM analysis; **A.** PALM images, center of peaks are shown. **B.** and **C.** Peaks in PALM images were grouped using the maximum blinking time of 25.6 s for Cage 552 and a group radius of 2.5 σ.
